# ASD–Time for a paradigm shift

**DOI:** 10.3389/fpsyt.2022.956351

**Published:** 2022-07-22

**Authors:** Yonata Levy

**Affiliations:** Psychology Department, Hadassah Medical School, Hebrew University, Jerusalem, Israel

**Keywords:** ASD, continuum, neurodevelopmental disorders, paradigm shift, meta-terms

Problems with the diagnosis of ASD have been acknowledged by clinicians and researchers alike. In a seminal paper in 2006, Happe et al. (1) called for the fractionation of autism arguing that the parameters by which autism was diagnosed at the time could not have a single explanation. Although the evidence has been there (2), side by side with worries expressed by clinicians, it took years for the implications of this work and many that followed to be digested and brought to the center of attention of research on ASD. Since Waterhouse and Gillberg (3) in which concerns about the categorical diagnosis of ASD were clearly expressed, the discussion took a more radical turn and a call to abandon the diagnosis of autism has been on the table. Finally, the last few years have seen more intensive work arguing for a reconceptualization of ASD, although still somewhat hesitantly. It seems we are still far from a consensus on the need for a paradigm shift with respect to ASD (4–6).

Of relevance to the current discussion on the status of ASD as a biological entity are writings of philosophers of science that concern the epistemological standing of psychiatric disorders, among them ASD. It is of course beyond the scope of this paper to do full justice to this topic. However, framing our discussion in reference to the terminology used by philosophers could help us see what is being claimed with respect to psychiatric disorders and whether the way ASD is defined in DSM V respects these conceptual boundaries.

Zachar and Kendler (7) review the history of psychiatric nosology tracing today's “crisis of confidence” (Ibid p. 50) to attempts in the 17^th^ and 18^th^ centuries to classify medical conditions and define their nature. They identify two positions in current day debate. The first involves gradual iterative improvement of DSM nosology. DSM-V has adopted this approach and has implemented a hybrid model of dimensions and categories in a number of psychiatric disorders including ASD (8). The second approach involves a paradigm shift, as exemplified in the RDoC initiative (9). RDoC severs ties between clinical and research constructs, organizing research around symptoms, not syndromes. Research domains in the RDoC model are anchored in behavioral neuroscience and cognitive theory, with the hope of increasing chances of discovering etiologies of psychiatric symptoms.

Kendler (10) discusses three theories about the nature of psychiatric disorders that can be placed on a scale of “realness,” namely, realism, pragmatism and constructivism. The latter are also referred to as “practical kinds” (7). Realism assumes that the content that comes under a diagnosis exists in the world independent of scientists' conceptions and activities. Natural kinds are “real”–they are bounded, stable and unified by virtue of a causal explanation. Much like a biological species, a “real” psychiatric disorder needs to be discovered, not created. Pragmatism sees psychiatric categories as a way of organizing practical aspects such as interventions, support systems and developmental predictions. To the pragmatist, reality is not a major concern. If an invented diagnostic category does the work, so be it. Constructivism does not give up realism, yet it accepts the fact that social-pragmatic concerns crucially affect the reality of psychiatric disorders. Thus, constructivism does not disjoin research and clinic. It welcomes the impact of social and cultural elements on the nosology, while aspiring to discover its biological essence.

Ongoing work in psychiatry has shown that realism as it is defined in the biological sciences sets prerequisites that cannot be met by psychiatric diagnoses. Still, giving up on the claim to “existence in the world” is a move likely to encounter objections from professionals in the field. Kemder's (10) limited view of realisms offers a working hypothesis that fits with the science of psychiatry, as well as with its clinical practices. In Kendler's words, a limited form of realism suggests that “a diagnosis is real to the degree that it coheres well with what we know empirically and feel comfortable about” (Ibid, p. 9). This is a narrower sense of realism that the field can and should adopt.

Genetic makeup, brain imaging, pathophysiology, developmental course, behavioral characteristics and treatment effects are parameters that provide the empirical basis of psychiatry. In line with the above suggestion of a limited version of realism, results related to these fields of study are expected to cohere as they relate to a given diagnostic category. If they do, they will confer a sense of reality on the projected entity. Does ASD as it is defined in DSM-V pass the test? and if it does not, is the field ready to reconceptualize ASD?

There seem to be three inter-dependent conditions that, if satisfied will lead to a reconceptualization of ASD. The first concerns a profound dissatisfaction among clinical and research communities as well as stakeholders with respect to the existing diagnosis of ASD. The second is the need to offer an alternative conceptualization that will get us closer to an understanding of the phenomena currently diagnosed as ASD and will meet patients' needs. The third involves the crosstalk among stakeholders. Few psychiatric diagnoses have had as much public impact as has been the case with ASD. For a new conceptualization to replace ASD, families, patients' associations, government support systems, social services, educators and funding agencies need to come to terms with a new way of thinking about ASD.

Is dissatisfaction with the current definition of ASD deep enough? The answer seems to be–Yes. Despite impressive technological progress and a growing understanding of brain structure and function, as well as the genetics of various developmental conditions, neurobiological research has not provided definitive answers that support a categorical definition of ASD. Our current understanding of genetic risk factors of neurodevelopmental disorders among them ASD suggests that they are polygenic, pleiotropic and are on a continuum with typical behavior (11). Polygenic variations seen on a large number of alleles jointly and probabilistically increased risk for a neurodevelopmental disorder. Beside risk alleles there are protective alleles as well as variations that improve performance, as is not rarely seen among people diagnosed with ASD (12, 13).

The genetic architecture of neurodevelopmental disorders overlaps. Risk alleles but also protective alleles have additive and overlapping effects that, in many cases can contribute to more than a single unique phenotype (11). Of particular relevance is recent evidence of a common factor, labeled the factor p, underlying diagnostically **diverse** developmental disorders, among them ASD (14). A recent study examined whether polygenic risk scores in school age children are associated with a general propensity for psychopathology or with specific disorder. The results suggest that phenotypes are more often associated with general pathology, rather than with one specific domain (15). In other words, questions about the validity of ASD as an independent category concern not only the heterogeneity within ASD but also the similarities across neurodevelopmental disorders. This conclusion is reinforced by neuroimaging studies of people with ASD, which present mixed results with few unique patterns that can be attributed to the diagnosis (16).

The message from neurobiological results as of now is the following: An individual's ultimate behavioral profile is a function of his/her genetic architecture, internal and external environmental effects, developmental history as well as stochastic events that interact to produce a behavioral phenotype. There seems to be no evidence for a DSM-type system of discrete categories that map onto psychiatric disorders, ASD included.

As for behavioral research, as early as 1971, acknowledging similarities and differences between children with different diagnoses, among them children with autism, Wing and Wing (2) stated that “a combination of language, perceptual, motor and autonomic impairments underlies autistic behavior... Such a combination could have a single or multiple etiologies. Isolated fragments of the full picture often occur, either alone or in combination with different syndromes” (Ibid p. 256). Fifty years of behavioral research confirmed this account.

Current diagnosis of ASD allows extreme within-category heterogeneity and lacks category-specific developmental course. Attempts to define sub-groups within the spectrum failed. Similar to studies in the biology of ASD, behavioral research typically fails to reproduce and the study population does not cover the entire spectrum. In particular, it fails to cover low functioning individuals as well as those without speech (17, 18). The phenomenon of regression is poorly defined, girls have been less studied than boys probably due to stereotyping and to biases in the diagnostic tools (19). Children in low-income countries are poorly represented in the studied populations (20) and this is the case with respect to adults with ASD as well (21). Finally, there is little predictive power relating to intervention effects on ASD symptomatology (22).

An extension of the problems inherent in the attempt to enclose the behaviors that characterize ASD within the boundaries of a labeled category is evident in the new category SCD (Social Communication Disorder). SCD is characterized in DSM-V as a communication disorder but is considered by some as a mild form of ASD (23, 24), as identical to what has been known in the literature as pragmatic language impairment (25), as a version of the BAP (26) or as providing motivation for an independent RRB category (27). Similar to ASD, SCD is framed in a DSM-type language. Much like ASD, the dimensional characteristics of SCD extending to typical children and adults, difficulties in its definition and its overlap with other language disorders (28) does not lend validity to SCD as a category.

In sum, results coming from diverse areas of study, all intensely researched in children and adolescents that have received a diagnosis of ASD, do not fulfill the limited sense of realism suggested by Kendler (10). That is, they fail to present a coherent picture that could convincingly define an entity. Rather, the observed phenomena are on continua with the distribution of similar behaviors in the typical population. In Hayman's (11) words, ASD, as well as other neurodevelopmental disorders. are “grounded in nature, but they are not natural kinds” (Ibid p. 21).

There is undoubtedly a sense of disappointment in the clinical and the research communities in having to admit that decades of work within a categorical framework of ASD, have resulted in “many insights, but few answers,” as stated in a recent review article on neuroscience research on ASD (16) (Ibid p. 4344). Nevertheless, many are reluctant to re-consider the categorical status of ASD. The reasons refer primarily to the worry that a re-conceptualization could affect patients' welfare on a variety of levels. Even more so, since there is no acceptable alternative against which the risks of such a move could be weighed (6). The second condition listed above, namely, the need to sketch a blueprint of a re-conceptualization of ASD, is therefore a most urgent task.

A new way of thinking about ASD may be inspired by the conceptualization of other neurodevelopmental disorders. Consider the following: the continuous nature of the behaviors diagnosed as ASD with behaviors seen in typical individuals, the overlap in the genetics of neurodevelopmental disorders, the frequent “comorbidity” with childhood syndromes, the potential for considering diagnosed individuals as diverse, not disordered–all of these characterize neurodevelopmental disorders, such as cognitive impairment or language impairment as well as ASD. Yet, neither cognitive impairment nor language disorders denote a diagnostic category. They are viewed as meta-terms, and are referred to in relation to DSM terminology as specifiers of a DSM diagnosis.

Are the defining parameters of ASD inherently and developmentally different from cognitive impairment or language impairment? Behavioral work tells us that this is not the case. In fact, they too are better described as meta-terms, on the same theoretical level as the currently-noted specifiers, such as cognitive level or language.

Note that dismantling ASD and reconceptualising each of its defining parameters as a meta-term that can have multiple behavioral manifestations and is not tied to a single diagnosis is not a semantic issue. This change gets us closer to the scientific truth, namely, to the picture that emerges out of the neurobiological, genetic and behavioral studies conducted on ASD in the past 50 years. It coheres well with what we know empirically and thus maintains a sense of realism (10).

Importantly, redefining ASD along these lines does not dissociate it from clinical terminology. Rather, it is a bottom-up approach, based on behavior and attentive to practical considerations, that has clinical advantages a well. In considering social-communication behavior, routine-repetitive behavior, cognitive impairment and language development, along with perceptual sensitivities, attention deficits and temperament as characterizing a child's profile relative to age and background, the within category dimensional approach is turned from vertical to horizontal, encompassing typical as well as atypical behavior. By adopting this approach, developmental science and the science of pathology may acquire a road map to variability, with respect to which it can resolve questions related to comorbidities and evaluate decisions as to needs and types of intervention. The lab and the clinic will definitely have a common language.

What about the prototypical cases of “pure” autism, described by Mottron (29)? I believe it is an open question whether such “pure” cases are instances of a diagnostic category. Assuming a consensus can be reached among expert clinicians with respect to a sufficiently large group of children who will be considered exemplars of “pure” autism, the existence of biological underpinnings of such a group could be tested. The possibility exists however, that “pure” autism, just like less prototypical cases, is the outcome of interactions among the polygenic factors and environmental effects that are involved in these set of behaviors. Note however, that the logic behind Mottron's hypothesis suggests that even if a unique causal, biological basis for the symptomatology that characterizes “pure” autism is found, it will not generalize to ASD as it is defined in DSM V. Thus, the need to reconceptualize ASD will remain.

Perhaps the major obstacle to an open discussion relating to the status of ASD is the third condition listed above, namely, the crosstalk among stakeholders. In the case of ASD it involves not only the clinic and the laboratory but the media, the public, the educational system, welfare and research funding. In the case of ASD, these are powerful social-political institutions whose position with respect to the controversy within academic and clinical quarters has not been heard yet. Constructivism tells us that nosology, formulated by the professional community, affects social organizations, resources and trends and is affected by them (10). Given this mutual dependency, the question whether we should reconceptualize ASD, must take into consideration these social factors as well.

In conclusion, despite concerns and difficulties, I believe it is the duty of the professional community to revolutionize ASD definition, aspiring for a conceptualization that will cohere with what research in relevant domains has taught us. We owe it to our patients and to the public. Given the current advancement in technological solutions, opportunities for big data analyses, network perspectives (30) machine learning methods (31), it seems that reliance on categories as systematizers of our knowledge base could become more relaxed, perhaps even obsolete. Medicine and psychology may face real-world considerations, such as suitable interventions, educational placement, and welfare without the aid of categorical labels. Such an approach will better connect clinical work and scientific research. Developmental psychiatry may be able to more effectively join other areas of medicine and apply personalized medicine successfully.

True, in the absence of a label, ASD may lose its public prominence, but hopefully, this will open up opportunities for children with other neurodevelopmental disorders or monogenic syndromes. Perhaps the public will turn its attention to them as well.

## Author contributions

The author confirms being the sole contributor of this work and has approved it for publication.

## Conflict of interest

The author declares that the research was conducted in the absence of any commercial or financial relationships that could be construed as a potential conflict of interest.

## Publisher's note

All claims expressed in this article are solely those of the authors and do not necessarily represent those of their affiliated organizations, or those of the publisher, the editors and the reviewers. Any product that may be evaluated in this article, or claim that may be made by its manufacturer, is not guaranteed or endorsed by the publisher.
